# Effect of Different Characters of the Pitcher Trap Syndrome in *Nepenthes* on Insect Trapping Efficiency: A Biomimetic Approach

**DOI:** 10.3390/biomimetics11030180

**Published:** 2026-03-03

**Authors:** Elena V. Gorb, Meike Lange, Anna Jamke, Stanislav N. Gorb

**Affiliations:** Department of Functional Morphology and Biomechanics, Zoological Institute, Kiel University, D-24098 Kiel, Germanysgorb@zoologie.uni-kiel.de (S.N.G.)

**Keywords:** adhesive pad, carnivorous plant, claw, coating, contact angle, digestive fluid, *Drosophila melanogaster*, epicuticular wax, microparticles, *Nepenthes alata*, surfactant

## Abstract

The aim of our study was to determine the importance of different pitcher syndrome characters (size of the trap, the presence of inner microscopic surface coverage, physical properties of the pitcher fluid) for insect trapping efficiency using artificial, “biomimetic” pitchers. We performed trapping experiments with *Drosophila melanogaster* flies, applied cryo scanning electron microscopy for characterization of the topography of surface coatings and visualization of their contaminability effects on insect attachment organs, and conducted contact angle measurements with different liquids used in experiments. The type of the liquid used as the pitcher fluid had the most important impact on the trapping efficiency; surfactant-containing liquids exhibiting strong wetting properties provided a high number of trapped flies. The diameter of the trap rather than its height influenced insect trapping efficiency; apparently, because wider traps provide a larger space for more insects to get into a trap, they captured more flies in comparison to narrower traps. The presence of both the calcium carbonate and kaolin coatings mimicking the epicuticular wax coverage inside pitchers in many *Nepenthes* species additionally contributed to the trapping success due to a reduction of contact between insect feet and the trap surface and to contamination of flies’ attachment organs by detached microparticles.

## 1. Introduction

Carnivorous plants, usually called insectivorous plants, are distributed worldwide and comprise more than 860 species from 18 genera of angiosperms growing commonly in nutrient-poor habitats [1,2,3]. In addition to photosynthesis, these plants rely on capturing small animals, typically arthropods (mostly insects), and possess a cluster of characters and mechanisms (traits) that altogether build up the carnivorous syndrome, ensuring the following six main functions: (1) to attract, (2) retain, (3) trap, (4) kill animals, (5) digest the captured prey, and (6) absorb the derived nutrients [4,5]. All of this is achieved by the use of specialized trapping organs (in most cases, transformed and specialized leaves)—either passive ones that do not employ movement (pitfall, lobster pot, passive flypaper) or active motile ones that apply various movements to catch prey (active fly paper, snap or steel trap, mousetrap) [4,5]. Newly, a passive motile trapping mechanism, which is based on a rain-actuated ‘springboard’ movement of one part of the passive trap, was discovered [6,7,8].

Among passive pitfall traps, which are gravity-driven and characteristic of Nepenthaceae, Sarraceniaceae, Cephalotaceae, and some Bromeliaceae, pitchers of tropical pitcher plants *Nepenthes* L. (nearly 200 species according to [9]) (Nepenthaceae) are classical examples of such trapping organs. Most representatives of this Palaeotropic genus are vines or subscandent shrubs, typically growing terrestrially or in a few cases epiphytically [10]. All species produce pitchers, usually of two types (i.e., are dimorphic): ovoid/globose terrestrial (lower) ones bearing wing-like structures, which contact the ground, and funnel-shaped/cylindrical aerial (upper) ones lacking “wings” [10,11]. Whereas lower pitchers are developed by young immature plants, upper ones appear when plants reach a certain age [12]. Interestingly, it has been found that in some *Nepenthes* species, different types of pitchers are specialized in capturing different prey taxa [13,14,15,16,17]. 

**Figure 1:**
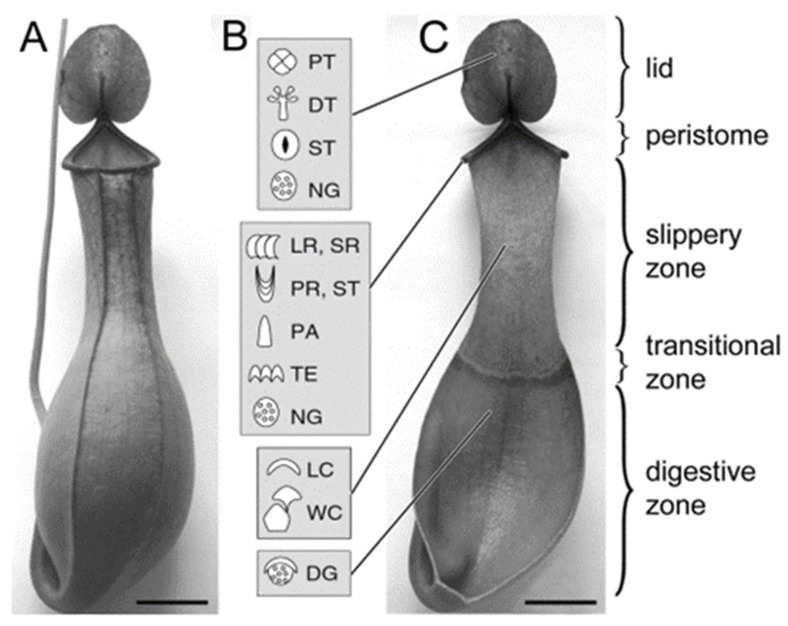
*Nepenthes alata* Blanco pitcher: general view (**A**); dissected pitcher with functional zones (**C**); schematic representation of surface structures characteristic for each zone (**B**). Scale bars: 2 mm. DG, digestive glands with hoods; DT, dendroid trichomes; LC, lunate cells; LR, large ridges of the first order; NG, extrafloral nectar glands; PA, papillae; PR, parabolic ledges; PT, peltate trichomes; SR, small ridges of the second order; ST, stomata with guard cells; TE, teeth of the inner arm margin; WC, wax projections. Adapted from [26].

Developed at the tips of the tendrils coming from the tips of the leaf blades (Figure 1A), *Nepenthes* pitchers have a zoned structure on the inner surfaces and can be divided into several regions (zones), which differ in both morphology and presence of specialized microstructures and are related to different functions (Figure 1B,C) [4,18]. The lid and peristome, a collar-shaped structure around the pitcher mouth, play the main roles in prey attraction primarily due to numerous extrafloral nectaries (Figure 1B,C) and comprise the so called “attractive zone” (according to [4]), although also their coloration pattern [4] and the presence of the peculiar tomentose tissue near the peristome [19,20] can contribute to the attraction of prey animals in particular *Nepenthes* species. Additionally, the lid not only covers the pitcher mouth and offers shelter from heavy rain [21,22] but may also assist in prey capture due to the downward orientation, which affects the attachment negatively [23]. Presently, it was found that rain drops can actuate the ‘springboard’ movement of the lid in *N. gracilis* Korth. and *N. pervellei* Blume that catapults insects into the trap [6,7,8,24]. As for the peristome, recent experimental studies have revealed its key role in prey capture. Due to combination of the hydrophilic surface chemistry, overlapping and anisotropic arrangement of epidermal cells, specific surface roughness, and the presence of hygroscopic nectar, the peristome achieves extremely high wettability and slipperiness, which cause insects to slip by hydroplaning on a thin liquid film and to fall down inside the pitcher [22,23,25].

The second zone previously called a “conductive, slippery zone” [4,18,27,28] is characterized by the presence of lunate cells (modified stomatal guard cells) and epicuticular wax coverage on the inner pitcher wall (Figure 1B,C). It is often referred to as a “wax-coated”, “wax secreting”, or “waxy” zone (e.g., [4,21,29]). The lunate cells are responsible for surface anisotropy; insects easily move downward, in the direction of the pitcher bottom, but cannot interlock with their claws to concave, down-oriented hoods of lunate cells and, therefore, are not able to climb upward and escape from the pitcher [4,30,31,32]. The wax coverage, which is composed of one or more superimposed layers of wax platelets protruding perpendicular from the pitcher wall (e.g., [26,28,33,34]), reduces insect attachment primarily due to the presence of critical surface microroughness created by wax particles [33,35] and contamination of insect attachment organs by detached particles [21,29,30,32,33,36]. It has been also suggested that since the wax coverage exhibits highly hydrophobic properties and low free surface energy [26,30,37,38], it minimizes insect attachment due to very low capillary forces. Previously, this pitcher zone was considered to play the main role in prey capture (e.g., [4,21,30]), whereas recently, it is believed to be associated principally with the retention of prey and only secondly—with the initial trapping. However, comparative morphological studies of two pitcher zones primarily relevant to prey catching (the peristome and slippery zone) found (1) different types of slippery zones (with well-developed two-layered wax coverage, with greatly reduced wax coverage composed of solitary wax protrusions, and without wax) and (2) significant negative correlation between the length of the slippery zone and the width of the peristome [34,39]. Based on the analysis of the relationship between pitcher macromorphology and microstructure of the slippery zone, two types of *Nepenthes* pitchers have been proposed according to the main trapping mechanism: (1) based predominantly on the slippery waxy zone and (2) based on the peristome.

The next zone lacking any special surface structures is attributed as a “transitional zone” between the waxy and digestive zones (Figure 1C) [34] and is functionless according to [4].

The deepest zone inside *Nepenthes* pitchers, which bears numerous digestive glands, is called “digestive” or “absorptive” (Figure 1B,C) [4]. It is mainly associated with prey utilization—secretion of digestive enzymes and other molecules related to prey digestion, absorption, and transport of gained nutrients (e.g., [4,40,41,42,43,44,45]). Although not serving as a specialized trapping zone, the digestive zone can hinder the escape of insects from the pitcher; downward-directed epidermal lips that partially shield digestive glands do not provide a foothold for insect claws similar to the lunate cells of the slippery zone [4,18], and the secretion covering the surface inhibits attachment of insect pads [4]. Later experimental studies on *N. alata* and *N. × ventrata* Hort. ex Fleming revealed the contribution of this zone to the retaining function of the pitchers [30,46]. Firstly, frictional anisotropy of the glandular zone in terms of its influence on insect attachment was proven, and secondly, the adhesive (“gluing”) effect of the glandular secretion, at least at adhesive areas on the surface, impeding the locomotion of some insects was demonstrated.

The digestive fluid inside the pitcher, besides its important role in prey digestion, serves to coat, suffocate, and quickly drown animals downfallen onto the fluid surface, which is surface active [4]. Moreover, according to several recent studies, the pitcher fluid in some *Nepenthes* species exhibits certain viscoelastic properties that prevent insects from pulling themselves out of the fluid [15,47,48]. It has been shown for *N. rafflesiana* Jack that the pitcher fluid viscoelasticity plays a more important role in prey retention compared to the slippery zone (both lunate cells and epicuticular waxes) [15,47]. It has also been suggested that the digestive fluid for prey capture (retention) in *Nepenthes* species lacking a slippery zone or slippery wettable peristome is fundamentally important [15,47]. Interestingly, based on the investigation of 23 *Nepenthes* species, Bonhomme et al. [48] found an inverse relationship between the viscoelasticity of the digestive fluid and the amount of wax produced in pitchers, suggesting the predominance of one of these two trapping mechanisms depending on the plant species, growing conditions, and type of prey. Recently, it has been reported that the high acidity of the digestive fluid can also contribute to the pitcher’s trapping success [17,49].

Thus, there is a great amount of older and recent literature suggesting, examining, and proving the roles of different characters of the carnivorous syndrome in prey capture by *Nepenthes* pitchers, which is presented above, as well as a number of rather new publications in main or partly reviewing different aspects of trapping mechanisms in these pitchers (e.g., [8,10,12,17,45,50,51,52]). In particular, it has been found that the use of various trapping mechanisms (peristome-based, wax-based, or viscoelastic-fluid-based) is driven by climate: *Nepenthes* species with peristome-based and viscoelastic fluid-based mechanisms are associated with humid conditions, where the viscoelastic-group of species has the most restricted area, whereas the wax-based mechanism is employed in both humid and more seasonal regions [51]. The comparative study of the pitcher characters and prey composition in seven *Nepenthes* taxa from the same natural habitat has demonstrated that generalist carnivorous species had a sweet odor, wide pitcher opening, and acidic pitcher fluid, whereas different combinations of morpho-functional traits (capture syndromes) were developed for prey specializations, such as (a) extrafloral nectar production, fluid acidity, and slippery waxy walls for ant trapping, (b) narrow cylindrical pitchers, rim of edible trichomes or symbiotic association with ants, and non-viscous fluid for termite capture, and (c) odor, funnel-shaped pitchers, large pitcher opening, and acidic viscoelastic fluid for flying insects [17]. Later, based on functional traits present in their pitchers, *Nepenthes* species have been classified into 12 different functional types, with a fundamental division between wax-bearing and wax-lacking species [12]. As an ancestral type, ovoid/cylindric pitchers with an apical, horizontal opening, clearly separated a basal, glossy, fluid-containing, digestive part and an upper matt, whitish or greyish part covered by microscopic wax structures have been proposed. A recent study on the springboard trapping mechanism [6,7,24] found in two *Nepenthes* species that requires three independent components, such as horizontal orientation of the lid, slippery wax-bearing lower surface, and pivoting oscillations, showed that this new composite mechanism evolved not through directional selection in the component traits but arose convergently by “spontaneous coincidence” of a new beneficial trait combination [8].

The aim of our study was to determine the importance of different pitcher syndrome characters, such as the size of the trap, the presence of inner microscopic surface coverage, and physical (wetting) properties of the pitcher fluid, for pitcher trapping success. For simplicity, we did not consider here either the pitcher peristome or the surface anisotropy characteristic for either the slippery waxy or glandular zone; their impacts on prey trapping and retention have been experimentally shown previously [23,25,30,31,32,46].

Previous experimental studies on the functional significance of different pitcher zones and their features in relation to prey capture were performed using native trapping organs, often with live plants, which limits the ability to systematically manipulate individual traits and assess their independent functional significance. In our study, for the first time, we applied artificial, “biomimetic” pitchers such as plastic tubes (lacking both the peristome and anisotropy in the inner pitcher surface), where we imitated different functionally significant pitcher characters. This approach made it possible to experimentally vary certain parameters in order to examine their importance for prey trapping. Such controlled biomimetic approaches that could disentangle the interactions between pitcher morphology, surface microstructure, and fluid properties are largely lacking, preventing a mechanistic understanding of how each feature contributes to prey capture and retention. A similar methodology of mimicking biological structures for a deeper understanding of functional principles of biological systems has been used in previous research on epicuticular plant wax coverage [53], animal attachment devices [54], and insect wings [55].

Taking into account our previous extensive morphological, ultrastructural, and experimental studies performed with *N. alata* [26,29,30,31,33,36,37,38,46], we selected this tropical pitcher plant species as a native prototype for our biomimetic pitchers. We defined *N. alata* pitchers as the ancestral type according to [12]. In nature, *N. alata* plants grow in mossy forests at 800–2400 m altitude on several Philippine Islands [9,11]. The plants produce both types of pitchers (lower when young and upper when mature), which do not differ much from each other.

Our working hypothesis was that a tube partly filled with fluid is able to trap insects (i.e., functions as a primitive trap), whereas the particular tube shape and inner surface coverage additionally increase the trapping efficiency. Trapping efficiency (trapping success) is defined here as number of prey items captured per tube or pitcher. To test this, we performed several sets of trapping experiments with *Drosophila melanogaster* (Meigen, 1830) flies (Diptera, Drosophilidae) and tubes (1) containing different liquids (water, Triton X-100, or Domol), (2) differing in dimensions (both height and diameter), and (3) having different microparticle coatings (calcium carbonate or kaolin). This fly species was chosen as a representative of small flying insects from the insect order Diptera, which is known to be one of the most frequently captured by the pitcher plants [4,13,17]. For the experiments, we considered that our artificial pitchers (tubes) did not function as traps if no or a very few insects were captured. Based on the results of some preliminary statistical exercises, starting from three trapped prey items equaling 1% of released insects, we assumed that these tubes can be regarded as trapping structures.

We also examined insect attachment organs in a clean condition and after flies contacted microparticle coatings. Furthermore, we estimated the wetting properties of liquids used in trapping experiments.

## 2. Materials and Methods

### 2.1. Trapping Experiments

**Figure 2:**
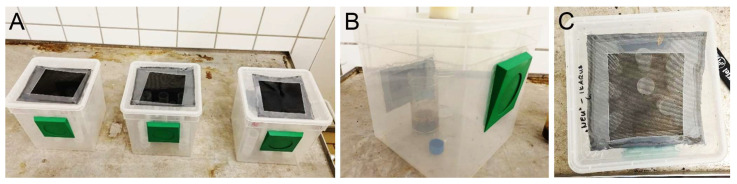
Plastic boxes used for experiments: boxes in the climate chamber (**A**); the box containing a tube used for the breeding of *Drosophila melanogaster* flies (**B**); the running experiment with different test tubes inside the box, view from above (**C**).

The experiments were performed in a climate chamber (Figure 2A) with a photoperiod of 14 light hours and 10 dark hours at a constant temperature of 25 °C and a relative humidity of 60%, where plastic boxes containing test tubes were placed (Figure 2C). A plastic box (19 cm × 19 cm × 19 cm, ca. 5.9 L; BraPlast, Vittsjö, Sweden) had a top covered with a fly screen and a flap door at the front, which was used to insert insects into the box (Figure 2).

**Figure 3:**
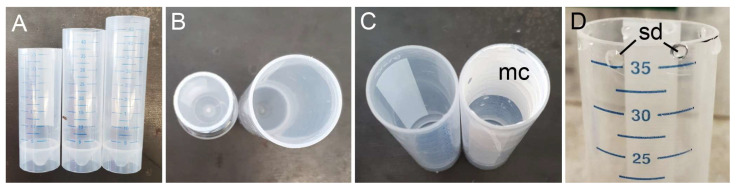
Plastic tubes used in the experiments: 30 mm in diameter and different heights (8, 9, and 10 cm, from left to right) (**A**); 10 cm high and different diameters (17 and 30 mm, left and right, respectively), view from above (**B**); tubes with intact (left) or modified, i.e., bearing a microparticle coating (right), inner surface, view from above (**C**); upper part of the tube with syrup droplets placed on its rim (**D**). For better visualization, the tube with a modified inner surface shown in (**C**) (right) has a wider strip of the coating than tubes used in the experiments. mc, microparticle coating; sd, sugar cane syrup droplets.

Two types of plastic (chemically inert, odorless, semitransparent polypropylene) cell culture tubes (CELLSTAR^®^ tubes, Greiner Bio-One, Frickenhausen, Germany) were used in the experiments: (1) 30 mm in diameter and with a 50 mL volume capacity and (2) 17 mm in diameter and with a 15 mL volume capacity (Figure 3B). The tubes were cut with a rotary carver to the right height needed for the experiment (8, 9, or 10 cm) (Figure 3A), and the resulting edges were polished by hand using sandpaper. The remaining polypropylene dust was cleaned off using compressed air. Such a procedure was applied to make all testing tubes.

To attract insects to the tubes, eight small 0.1 g drops of sugar cane syrup (Riemerschmid^®^, Erding, Germany) mimicking the nectar were placed on each tube rim using a pipette, four on its outer side and four on the inner side (Figure 3D). In experiments with the painted tubes, in order to avoid possible damage and/or contamination of a coating by the syrup, we positioned four droplets only on the outer tube side.

Test tubes were consistently filled to 20% of their volume with a liquid imitating the pitcher digestive fluid. This was either (1) water, (2) a water mixture of Triton X-100 in a 50:1 volume ratio (later called as Triton), or (3) a water mixture of 2.4.1. Domol heavy duty detergent in a 100:1 ratio (later called as Domol). Triton (4-phenyl-polyethylenglycol, Sigma-Aldrich, Hamburg, Germany) is a non-ionic surfactant and emulsifier. We used Triton from our old stock. However, because of its toxic effect, especially on humans and the environment, its use is now banned in the European Union (https://echa.europa.eu/authorisation-list, accessed on 1 April 2025). Therefore, in further experiments, we applied non-toxic Domol (Dirk Rossmann GmbH, Burgwedel, Germany), in which the main ingredients are surfactants (15–30% anionic and 5–15% non-ionic surfactants), including alkylbenzene sulfonates, sodium C12–18 sulphate, and linear alcohol ethoxylates C13 and C15.

The epicuticular wax coverage was simulated by two different microparticle coatings: (1) calcium carbonate and (2) kaolin. Three-centimeter-wide strips of the coating were applied to the upper part of the inner surface in the tube (Figure 3C). For the calcium carbonate (CaCO_3_) coating, the tube was painted using a brush with slaked lime (calcium hydroxide Ca(OH)_2_, Art-Nr. KK03.1; Carl Roth GmbH, Karlsruhe, Germany) dissolved in double-distilled water (20 g lime: 25 mL water) according to [56]. After air-drying, in the presence of carbon dioxide CO_2_, a microrough layer of calcium carbonate crystals was formed on the surface. Calcium carbonate is one of the first minerals used in the so called “ancient particle film technology” and represents insecticidal material, which has been widely applied in agriculture in the early 1900s and is still used nowadays against insect pests and plant pathogens [57]. The kaolin coating was obtained by painting the tube surface with a water suspension of kaolin powder (bolus alba = kaolin, Art-Nr. 8361.2; Carl Roth GmbH, Karlsruhe, Germany) containing mostly kaolinite, i.e., hydrated aluminum silicate Al_2_Si_2_O_5_(OH)_4_, in the ratio 1:1. After drying under air conditions for 24 h, a microstructured kaolin coating was created. Kaolin particle films along with other particle films, especially those based on aluminosilicates, are intensively used in agriculture for plant protection against insect pests, particularly in the last two decades [57]. The above two coatings differed in the shape and dimensions of their particles as well as roughness; the mean roughness (Ra) was 2.16 ± 0.44 µm for calcium carbonate vs. 1.54 ± 0.09 µm for kaolin, and the root mean square of roughness (r.m.s.) was 2.77 ± 0.61 µm for calcium carbonate vs. 1.93 ± 0.01 µm for kaolin [58,59]. For comparison, the wax coverage (upper layer) in *N. alata* pitchers showed Ra = 1.91 ± 0.25 µm and r.m.s. = 2.38 ± 0.30 µm [29]. All above roughness data were obtained at the same (×50) magnification using a white light interferometer.

The following experimental set-ups were realized to study different effects, such as

(a) Digestive fluid and (b) pitcher dimensions for

▪ Diameter:
  (1) Tubes 17 mm vs. 30 mm in diameter (both 10 cm high) filled with water;
  (2) Tubes 17 mm vs. 30 mm in diameter (both 10 cm high) filled with Triton;
  (3) Tubes 17 mm and 30 mm in diameter (both 10 cm high) filled with Domol;
• Height:
  (4) Tubes 8 cm vs. 9 cm vs. 10 cm high (all 30 mm in diameter) filled with water;
  (5) Tubes 8 cm vs. 9 cm vs. 10 cm high (all 30 mm in diameter) filled with Triton;
  (6) Tubes 8 cm vs. 9 cm vs. 10 cm high (all 30 mm in diameter) filled with Domol;

(c) Coverage of the inner pitcher surface imitated by

• Calcium carbonate:
  (7) Tubes intact vs. painted (10 cm high, 30 mm in diameter), filled with surfactant;
• Kaolin:
  (8) Tubes intact vs. painted (10 cm high, 30 mm in diameter), filled with surfactant.

Nine (3 + 3 + 3) or ten (5 + 5) tubes, depending on the experimental set-up (nine in (4)–(6) and ten in (1)–(3), (7), and (8)), were put in the random order into the plastic box, where 100 flies of *D. melanogaster* (the wild type *w118* obtained from the working group of Prof. T. Roeder (Department of Molecular Physiology, Zoological Institute, Kiel University)) were released. After 24 h, the number of flies trapped by the fluid in each tube was counted. We performed three experiments (in three boxes) simultaneously for every experimental set-up (Figure 3A). For analyses, results of the three repetitions were cumulated.

The number of flies trapped in different tube types was statistically analyzed for each experimental set-up using the Chi-square test (software Good Calculator, free online calculators, https://goodcalculators.com/). For each test, the expected number of trapped flies in each tube type was calculated as *expected = total number of trapped flies/number of tube types*.

To estimate whether the test tube type represented a trap, we calculated the average number of trapped flies per tube of this type for each experimental set-up: *average number of trapped flies per tube of this type = number of flies trapped by tubes of this type/number of tubes of this type*. If this number exceeded 3 (i.e., 1% of insect individuals inserted into the 3 boxes), this tube type was considered a trapping unit.

### 2.2. Cryo Scanning Electron Microsopy (Cryo SEM)

In order to visualize the possible contamination of insect tarsal attachment organs by microparticles, *D. melanogaster* flies (the wingless mutant *wg/Wnt-1* obtained from Prof. T. Roeder working group) were investigated after they had been kept for 1 h in (1) a clean non-coated tube, (2) a tube bearing a calcium carbonate coating, (3) a tube with a kaolin coating, or (4) an air-dried *N. alata* pitcher used as a control. The tested flies were then shock-frozen in liquid nitrogen (−196 °C) and prepared for cryo SEM examination as follows.

Insects were mounted with their backs to a holder and frozen in a cryo preparation chamber (ALTO 2500 cryo preparation system, Gatan Inc., Abingdon, UK) at −140 °C. Afterwards, they were sputter coated in a frozen condition with gold-palladium (6 nm thickness) and examined at a 3 kV acceleration voltage and a temperature of −120 °C at the cryo stage within the SEM Hitachi S-4800 (Hitachi High-Technologies Corp., Tokyo, Japan).

Additionally, the surfaces of both coatings and air-dried waxy zone of *N. alata* pitchers obtained from live plants growing in the greenhouse of the Botanical Garden at Kiel University were studied using the same cryo SEM approach.

### 2.3. Contact Angle (CA) Measurements

CAs of four different liquids (double-distilled water, Domol-water mixture, and Triton-water mixture used in the experiments as well as the digestive fluid from *N. alata* pitcher as a reference) were measured on five surface samples (smooth hydrophilic glass and hydrophobic Teflon, microstructured calcium carbonate and kaolin coatings, and the waxy zone of *N. alata* pitcher bearing the epicuiticular wax coverage as a control) using a high-speed optical CA measuring device OCAH 200 (DataPhysics Instruments GmbH, Filderstadt, Germany). We were primarily interested in the wetting properties of liquids used in the trapping experiments. The digestive fluid and pitcher samples were obtained on the day of measurements from live plants growing in the greenhouse of the Botanical Garden at Kiel University. We used 0.5 µL sessile drops and circle-fitting for the evaluation of static CAs. With each liquid on each surface, 10 measurements were performed. Data are presented in the text as the mean ± standard deviation.

Data on CAs obtained on different surfaces for each liquid were statistically analyzed using one-way ANOVA (for water and Triton) or Kruskal–Wallis one-way ANOVA on ranks (for Domol and the pitcher fluid) (SigmaStat 3.5, Systat Software Inc., Point Richmond, CA, USA), followed by the post hoc Holm–Sidak method (for water and Triton), Tukey test (for Domol) and Dunn’s method (for the pitcher fluid) for pairwise comparisons between samples. Data are presented in the corresponding figures as box-and-whisker graphs (https://en.wikipedia.org/wiki/Box_plot, accessed on 13 February 2026), where the box is drawn from the lower quartile Q_1_ (the median of the lower half of the dataset) to the upper quartile Q_3_ (the median of the upper half of the dataset) with a horizontal line inside it denoting the median Q_2_ (the middle value in the dataset). The whiskers are based on the 1.5 IQR value (IQR = Q_3_ − Q_1_): from Q_3_, a distance of 1.5 times the IQR is measured out and a whisker is drawn up to the largest observed data point from the dataset that falls within this distance; similarly, a distance of 1.5 times the IQR is measured out below the Q_1_ and a whisker is drawn down to the lowest observed data point from the dataset that falls within this distance. All other observed data points outside the boundary of the whiskers are plotted as outliers.

## 3. Results

### 3.1. Trapping Efficiency of Differently Prepared Tubes

Results of the trapping experiments performed according to eight different set-ups (1–8) (see Section 2. Materials and Methods, Section 2.1. Trapping experiments) are presented in Table 1.

#### 3.1.1. Effects of the Fluid and Tube Dimensions

In tubes filled with water (set-ups (1) and (4)), a very low number (less than 3 individuals per tube) of *D. melanogaster* flies were trapped. The highest amount of prey reaching in average 1.5 per tube was recorded in 10 cm high and 30 mm wide tubes (set-up (1)). In all, only 9 and 26 trapped flies (set-ups (4) and (1), respectively) were registered in these two experiments. However, at least one insect individual was trapped by each tested tube type.

In the case of water, tube dimensions have different effects on the trapping efficiency. Tubes with different heights (8, 9, and 10 cm, set-up (4)) captured a similar amount of prey (χ^2^ = 2.6660, d.f. = 2, *p* = 0.2636), whereas there was a statistically significant difference (χ^2^ = 15.3846, d.f. = 1, *p* < 0.0001) between tubes having different diameters (set-up (1)): 30 mm wide tubes trapped more flies than 17 mm tubes.

Compared to water-filled tubes, unpainted tubes filled with Triton demonstrated a higher quantity of trapped flies: 242 and 299 (set-ups (5) and (2), respectively) for every time 300 released flies were trapped in these two experiments. For each tube type, much more than three insects per individual tube, on average, were captured. The highest number of flies (14 and 16 in average in the set-ups (5) and (2), respectively) were trapped in 10 cm high and 30 cm wide tubes.

Both the length and diameter in clean, unpainted, Triton-filled tubes showed highly significant differences in terms of trapped insects: χ^2^ = 43.7654, d.f. = 2, *p* < 0.0001 for height (set-up (5)); χ^2^ = 109.2067, d.f. = 1, *p* < 0.0001 for diameter (set-up (2)). Among three different heights, the significantly best trapping success was achieved by the highest (10 cm high) tubes (*p* < 0.0001 for both comparisons), whereas shorter tubes (8 and 9 cm) did not differ from each other (*p* = 0.3964). In the case of diameter, better results were found in wider tubes.

Unpainted tubes filled with Domol trapped more *D. melanogaster* flies compared to those containing Triton and especially in comparison with water-filled ones; more than 280 flies were captured in each experiment. These tubes trapped on average from 9.5 (9 cm high and 30 cm in diameter, set-up (6)) to 16.0 insects (10 cm high and 30 mm in diameter, set-up (3)) per tube. The only exception was 17 mm wide tubes (set-up (3)), in which less than three insects (2.6 in average) were trapped.

The statistical comparisons between effects of the height and diameter in unpainted, Domol-filled tubes showed similar results to those found in water-filled tubes. Tubes of different heights (set-up (6)) trapped a similar amount of flies (χ^2^ = 2.4948, d.f. = 2, *p* = 0.2872), while tubes having different diameters (set-up (3)) demonstrated significantly different trapping efficiencies (χ^2^ = 143.2695, d.f. = 1, *p* < 0.0001), where 30 cm wide tubes were much more efficient.

#### 3.1.2. Effect of Microparticle Coatings

In both experiments with surfactant-containing tubes having microparticle coatings inside, all (calcium carbonate, set-up (7)) or nearly all (kaolin, set-up (8)) of 300 flies were captured. For both coatings, quantities of trapped insects essentially exceeded an average of three individuals per tube in both tube types tested (painted and unpainted), with lower numbers in unpainted tubes and higher numbers in painted ones.

Statistical analyses of fly numbers captured in different tube types detected significant differences between unpainted and painted tubes in both experiments with coatings: χ^2^ = 8.3334, d.f. = 1, *p* < 0.05 for calcium carbonate (set-up (7)) and χ^2^ = 97.6352, d.f. = 1, *p* < 0.0001 for kaolin (set-up (8)). Interestingly, when we compared the portions of trapped insects in unpainted and painted tubes, these values differed between coatings: it was 1.0:1.4 in experiments with calcium carbonate and 1.0:3.7 in experiments with kaolin.

### 3.2. Contamination of Insect Tarsal Attachment Organs by Different Microparticle Coverages

**Figure 4:**
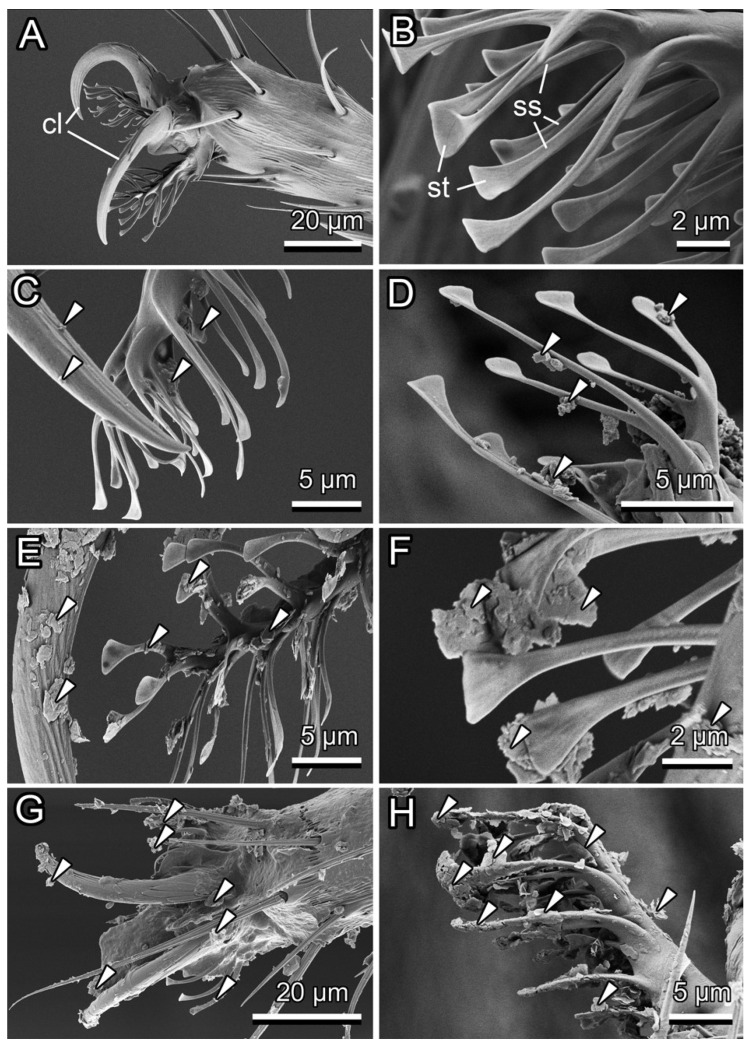
Cryo SEM images of *Drosophila melanogaster* attachment organs after experiments on clean tubes (**A**,**B**), tubes bearing the calcium carbonate (**C**,**D**) and kaolin (**E**,**F**) coatings, and air-dried pitchers of *Nepenthes alata* (**G**,**H**). White arrowheads point to the contaminating material. cl, claw; ss, shaft of tenent seta; st, tip of tenent seta.

Attachment organs (both claws and two hairy adhesive pads (pulvilli) bearing tenent setae) of *D. melanogaster* flies kept in non-coated tubes remained clean after the experiment (Figure 4A,B).

**Figure 5:**
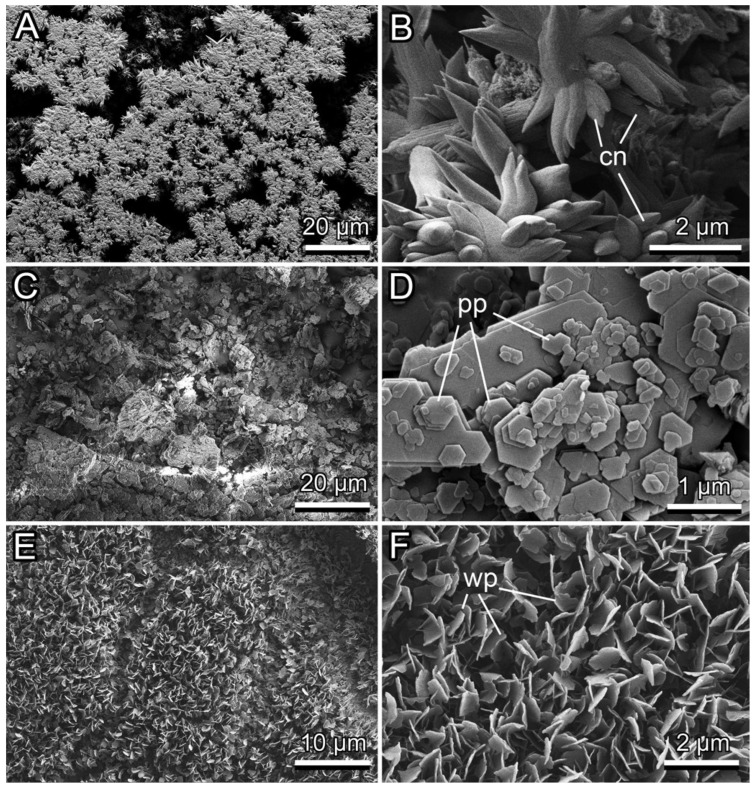
Cryo SEM images of the surfaces with the calcium carbonate (**A**,**B**) and kaolin (**C**,**D**) coatings and the epicuticular wax coverage inside the *Nepenthes alata* pitcher (**E**,**F**). cn, cone-shaped structure; pp, polygonal platelet; wp, wax platelet.

The calcium carbonate coating represented a rather loose coverage composed of separate flower-shaped groups, like rosettes, of cone-shaped structures, which were oriented at various angles to the underlying surface (Figure 5A,B). Contact with this coating inside the tubes led *D. melanogaster* to very little contamination of the adhesive pads, in a few cases (Figure 4C,D). If present, the contaminating material was located mainly on shafts of tenent setae or stuck between them. Weak contamination was also visible on claws (Figure 4C). These contaminating particles had irregular compact shapes.

The kaolin coating was also not compact and consisted of several superimposed layers of polygonal platelets placed parallel to the underlying surface (Figure 5C,D). This coating caused rather strong contamination of both claws and adhesive pads by relatively big flattened patches (Figure 4E). In addition to setal shafts and bases, spatula-shaped setal tips were dirty (Figure 4F). In some cases, setal tips stuck together due to the contaminant between them.

The surface of the epicuticular wax coverage of the slippery zone inside the *N. alata* pitcher had a rather uniform appearance due to densely and regularly placed, perpendicularly protruding platelet-shaped wax projections (Figure 5E,F). The experiments with *N. alata* wax coverage resulted in a very heavy contamination of the whole fly foot with flat wax particles (Figure 4G,H), where the adhered wax material often caused the conglutination of the setae (Figure 4H).

### 3.3. CAs of Tested Liquids on Different Surface Samples

In cases of both microparticle coatings (calcium carbonate and kaolin), all tested liquids were immediately sucked in, i.e., absorbed. In these cases, we regarded CAs as 0°.

**Figure 6:**
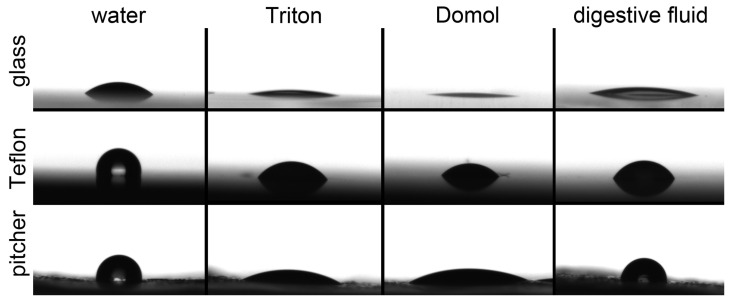
Exemplary 0.5 µL droplets of four liquids (water, Triton, Domol, and the digestive fluid of the *Nepenthes alata* pitcher) on three surface samples (glass, Teflon, and the waxy surface of the *N. alata* pitcher).

Three liquid-surface-air systems showed CAs exceeding 90° (Figure 6). These were water on the waxy pitcher surface of *N. alata* (103.84 ± 5.96°), water on Teflon (100.83 ± 5.70°), and the digestive fluid of the pitcher on the waxy pitcher surface of *N. alata* (95.09 ± 11.65°). In other tested systems, CAs ranged on average from around 7° (Triton on glass) to almost 59° (the digestive fluid on Teflon).

**Figure 7:**
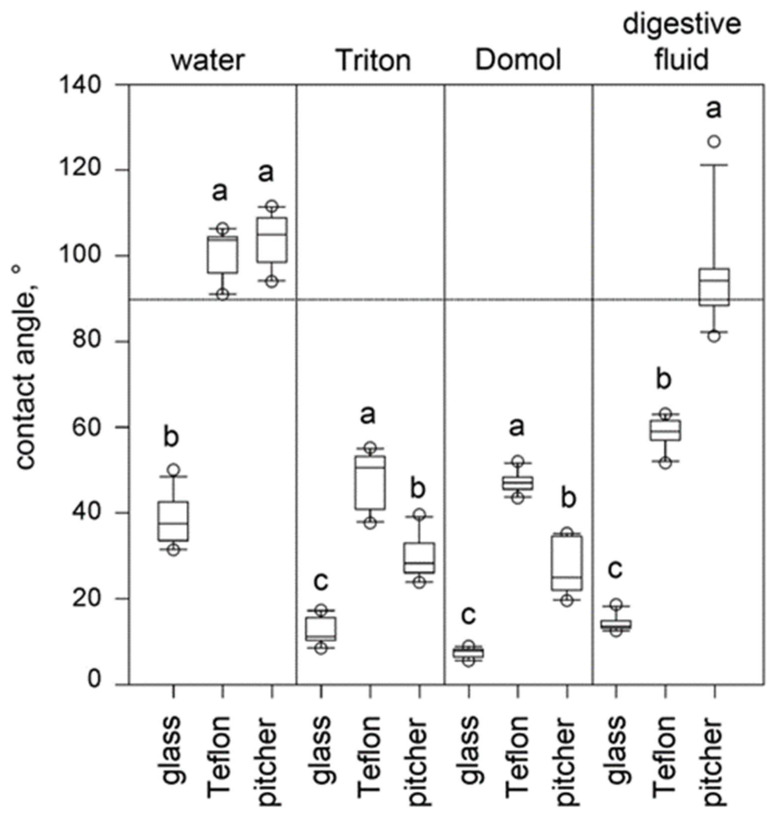
Wetting properties of four different liquids. Boxplots show the interquartile range and the medians, whiskers indicate the 1.5× interquartile range, and “◦” are outliers. Data represented by boxplots with different letters within one liquid are significantly different at *p* < 0.05. A dashed line marks 90°.

Figure 7 shows the wetting properties of four tested liquids on three solid surface samples. Water demonstrated significantly different CAs on the test surfaces (one-way ANOVA, F = 461.815, d.f. = 2, *p* < 0.001). It readily wetted glass samples, whereas Teflon and the waxy pitcher surface had significantly similar hydrophobic properties (Holm-Sidak method: glass vs. Teflon and glass vs. pitcher, both *p* < 0.05; Teflon vs. pitcher, *p* = 0.253). Triton and Domol showed similar wetting properties; all tested surfaces were wetted by both liquids, however, with significantly different CAs (one-way ANOVA for Triton, F = 131.346; Kruskal–Wallis one-way ANOVA on ranks for Domol, H = 25.806; both d.f. = 2, *p* < 0.001): the lowest CAs on glass, the highest ones on Teflon, and the intermediate ones on the pitcher surface (Holm-Sidak method for Triton and Tukey test for Domol, *p* < 0.05 in all comparisons). As for the digestive fluid, both glass and Teflon were wetted, although with significantly different CAs, whereas the waxy pitcher zone was unwettable (Kruskal–Wallis one-way ANOVA on ranks: H = 26.673, d.f. = 2, *p* < 0.001; Dunn’s method, *p* < 0.05 in all comparisons).

**Figure 8:**
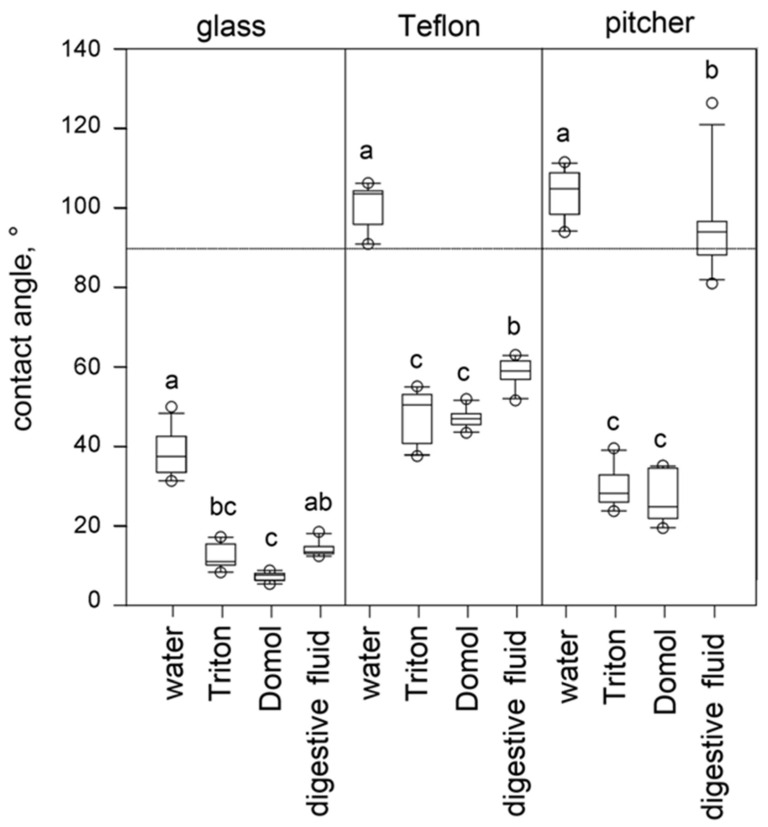
Wettability of three surface samples. Boxplots show the interquartile range and the medians, whiskers indicate the 1.5× interquartile range, and “◦” are outliers. Data represented by boxplots with different letters within one surface sample are significantly different at *p* < 0.05. A dashed line marks 90°.

Figure 8 shows the wettability of three solid surface samples by four different liquids. Glass was well wetted by all used liquids, much better than Teflon and the pitcher surface (Kruskal–Wallis one-way ANOVA on ranks for all, H = 117.421, d.f. = 11, *p* < 0.001; Dunn’s method for glass vs. Teflon and glass vs. pitcher surface, *p* < 0.05 in all comparisons). The latter two surfaces demonstrated the same hydrophobic properties (Dunn’s method, *p* > 0.05) and were rather similar, although with significantly different CAs, in cases of Triton and Domol (Dunn’s method, both *p* < 0.05) but showed completely different behaviors in regard to the pitcher fluid; Teflon was wettable (58.90 ± 3.32°), whereas the pitcher surface was unwettable.

## 4. Discussion

### 4.1. Effects of the Tube Dimensions, Inner Surface Coatings and Liquids on Insect Trapping Efficiency

Our trapping experiments clearly demonstrated that the type of liquid has a more important influence on the trapping efficiency compared to different tube dimensions. Although all tested tubes of different sizes containing water as an analogy to the pitcher fluid captured a very small number of insects, Domol-filled and especially Triton-filled tubes served as trapping structures capturing (much) more than 1% of released flies. Our CA measurements showed that in contrast to water, both surfactant-containing liquids (Triton and Domol) showed similar, strong wetting properties; they readily wetted all tested surfaces, among them hydrophobic Teflon and the waxy pitcher surface. Interestingly, the digestive fluid from *N. alata* pitchers demonstrated similar properties in the case of hydrophilic glass, still wetted Teflon, though at a higher contact angle, but was not able to wet the pitcher’s waxy surface. From results of our CA measurements, we could conclude that both Triton and Domol were liquids with similar physicochemical properties to the plant liquid and therefore proper to use as biomimetic fluids in our trapping experiments.

Our results on CA of water on the *N. alata* waxy pitcher zone are in line with those obtained in the previous investigation on the wettability of different inner pitcher surfaces in this plant species [37], where the waxy zone showed superhydrophobic properties and was also very poor wetted (CAs > 120°) by two other liquids, such as extremely non-polar diiodomethane (surface tension = 50.0 mN/m, dispersion component = 47.4 mN/m, polar component = 2.6 mN/m [60]) and polar ethylene glycol (surface tension = 48.0 mN/m, dispersion component = 29.0 mN/m, polar component = 19.0 mN/m [61]). It has also been previously demonstrated in the experiment with spraying water over the waxy zone of *N. alata* pitchers that water forms separate droplets having a very high contact angle with the surface [26,38].

The digestive fluid in *Nepenthes* pitchers contains many digestive enzymes, antimicrobial compounds, mineral nutrients, and acidic polysaccharides [47,52,62]. The role of this fluid in carnivorous pitcher plants has been mainly studied in terms of its contribution to prey utilization, namely its chemical function in digestion, absorption, and transport of the prey-derived nitrogen compounds. As for its trapping function, it has been suggested that the pitcher fluid in *N. alata* does not act as a paralyzing substance like it has been previously demonstrated for *Sarracenia flava* L. (Sarraceniaceae) pitchers [4] but rather as a kind of glue that impedes insects’ locomotion [30]. Later, it was found first in *N. rafflesiana* and then also in *N. ampullaria* Jack, *N. gracilis*, and *N. hemsleyana* Macfarl. that this fluid exhibits a good wetting capability and a low surface tension; it wets an insect as soon as motion occurs and makes escaping from the pitcher almost impossible [47,49]. In several studies, the trapping success of the digestive fluid has been concatenated mainly to its viscoelastic properties, especially its high viscosity [12,15,47,63]. It has been reported that pitchers of many other *Nepenthes* species produce digestive fluid having similar physicochemical properties [48,64]. Especially in wax-lacking *Nepenthes* species, the digestive fluid was found to be particularly viscous [64,65]. According to [17,49], also the acidic pH of the fluid contributes to prey capture. The recent experimental study revealed that (1) insects sink more easily in the *N. rafflesiana* pitcher fluid than in water due to its reduced surface tension, (2) within the pitcher fluid, more energy is required to separate insects from the fluid than from water, and (3) the fluid prevents dewetting and makes it difficult for insects to free themselves [66]. Thus, based on the results of various studies, the digestive fluid was considered to induce the trapping and retention of prey in *Nepenthes* pitchers. In our trapping experiments, two used liquids (Domol and Triton) contained surfactants and, therefore, were able to readily wet *D. melanogaster* flies. At least owing to these wetting properties, both test liquids essentially contributed to insect capture, in contrast to water.

The wetting behavior of tested liquids closely reflects their surfactant composition. Water, lacking surfactants, exhibited high contact angles on hydrophobic surfaces such as Teflon and the waxy pitcher zone, whereas Triton and Domol, containing non-ionic and anionic surfactants, readily spread across all surfaces, reducing contact angles and mimicking the low surface tension characteristic of natural digestive fluids. Notably, the digestive fluid of *N. alata*, despite being aqueous, displayed selective wettability (wetting hydrophilic surfaces while the waxy pitcher zone remained unwet), demonstrating that its complex composition of surface-active compounds achieves a combination of adhesion, slipperiness, and prey capture not fully replicated by simple surfactants.

The experimental data showed a certain difference between the effects of height vs. diameter of the tubes on their trapping function. Filled with water, tubes of different heights were equally inefficient in trapping, whereas wider tubes captured significantly more flies than narrower ones. In the case of Triton, both the height and diameter contributed to the prey capture: higher and wider tubes demonstrated significantly stronger trapping success. With Domol-filled tubes, we obtained similar results to those found in water-filled tubes, however, with a much higher number of trapped insects. These effects, which were especially pronounced in experiments with Triton-filled tubes, may have the following explanations. In the case of higher/slender tubes, the probability that insects fly out and escape is much lower because of the relatively high walls of the trap. The fact that we did not see the effect of the tube height in our tests with water- and Domol-filled tubes may be a result of a rather small range (8–10 cm) of the used variability. Possibly, when we apply stronger differences in height (i.e., 5, 7.5 and 10 cm), we would obtain more distinct differences in prey capture between tube types. As to the impact of the diameter, it may be explained by a straightforward geometrical effect: more insects could get into a wider trap. Previously, in a study with *Nepenthes* plants, it has been shown that pitchers having a larger diameter of pitcher opening captured more flying insects than those with a narrower one [17]. Our results obtained with wide tubes are to a certain extent in line with this finding. The previous authors suggested that fluid-filled funnel-shaped pitchers with a large diameter probably act as reflection–polarization traps attracting flying insects [17]. Since our experiments were performed with cylindrical tubes and the distance between the fluid level and tube opening was relatively long, we doubt whether a large diameter can make the fluid more visible to insects in this case.

**Figure 9:**
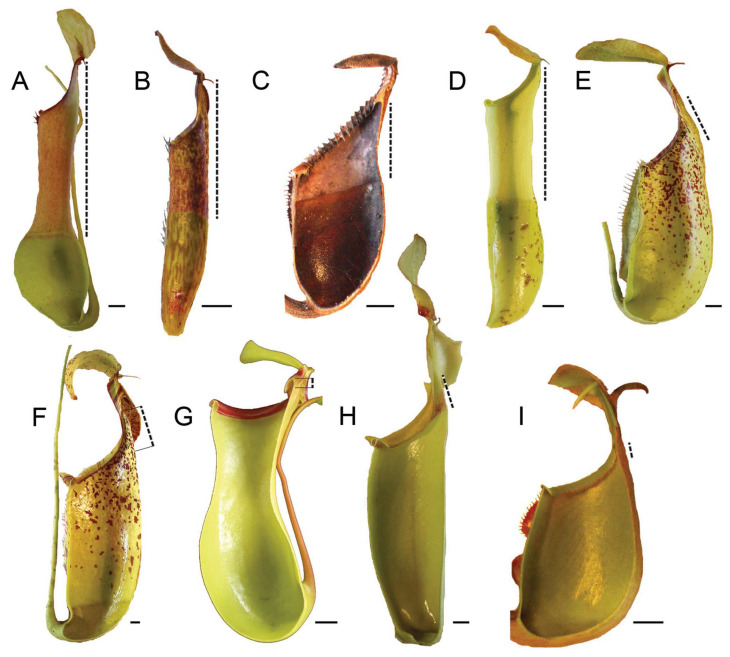
Longitudinally dissected pitchers of different *Nepenthes* taxa, view from inside: *N. alata* (**A**), *N. fusca* Danser (**B**), *N. macrophylla* (Marabini) Jebb and Cheek (**C**), *N. mirabilis* (Lour.) Druce (**D**), *N. rafflesiana* (**E**), *N. dicksoniana* Lindsay (horticultural hybrid of *N. rafflesiana* and *N. veitchii* Hook. f.) (**F**), *N. ventricosa* Blanco (**G**), *N. veitchii* (**H**), and *N. bicalcarata* Hook. f. (**I**). A dashed line denotes the length of the slippery (waxy) zone. Scale bars: 1 cm. Adapted from [34].

Although most *Nepenthes* species are known to vary in their prey composition, as they are usually not specialized in one species of prey but rather catch different arthropods in general [20], their pitchers still differ from each other in size, shape, and color pattern [52], depending not only on the plant species (Figure 9) but also on the location on the plant. Generally, when pitcher dimorphism is present, the lower pitchers are smaller, more compact, and overall wider than the upper pitchers, which prevalently prey flying insects, as described, for example, in *N. rafflesiana* [13] and *N. madagascariensis* Poir. [67]). In these cases, the lower pitchers better accommodated non-flying insects and provided more space for them to crawl into. Recently, in a study on seven *Nepenthes* species, it has been demonstrated that pitcher volume and diameter did not differ significantly between upper and lower pitchers, whereas upper pitchers, where trapped flying insect were more abundant, were, on average, higher and had a more conical shape [17]. Interestingly, the number of ants trapped in pitchers was similar in both pitcher types.

*Nepenthes alata*, which served as a kind of native prototype for our “biomimetic” pitchers, is a highly polymorphic climbing plant species, with pitchers varying in color (from green to red) and dimensions reaching 25 cm in height and 6 cm in diameter. Although shape does not change much between the lower and upper pitches, there are some differences in size; close to the ground, the pitchers are usually smaller (up to 18 cm high), whereas the upper ones reach 25 cm in height. In our trapping experiments, we only slightly varied the height of the tubes (8, 9, and 10 cm) and, therefore, probably did not detect any effect of the tube height in most experimental set-ups. As for the pitcher diameter, it does not vary in *N. alata*, but this can be observed in other *Nepenthes* species [65]. For this reason, in order to understand the influence of the diameter on prey capture, we used narrow (17 mm in diameter) and wide (30 mm in diameter) tubes in our study. Our experimental results clearly demonstrated that wider tubes are more efficient in trapping.

In contrast to previous conclusions that cylindrical pitchers with waxy walls are particularly efficient for ants’ capture in comparison to termites’ and flying insects’ capture [17] and wax only apparently is efficient for trapping ants [48], our experiments clearly demonstrated the effect of microscopic surface coverage on trapping flying insects. The use of a microparticle coating that simulated the epicuticular wax coverage inside the pitcher revealed its significance for the trapping function in the cases of both types of coatings (calcium carbonate and kaolin) and both surfactant-containing liquids (Triton and Domol). In these experiments, all or almost all inserted *D. melanogaster* flies were trapped. Interestingly, both intact and painted (i.e., bearing a coating) tubes successfully captured insects, however, in different proportions depending on the experimental set-up, namely the type of the coating and the type of the liquid; the kaolin coating in Domol-filled tubes trapped relatively more flies in comparison to calcium carbonate coating in Triton-filled tubes. The above microparticle coatings created a certain “critical” microroughness of the surface, which decreased the contact area between the surface and insect adhesive pads dramatically, leading to a reduction of attachment forces (according to [68,69,70]) and resulting in a higher trapping efficiency of coating-bearing tubes. Such an effect of the contact area reduction has been previously shown for the epicuticular wax coverage in native pitchers of some *Nepenthes* species [30,33,35]. Moreover, contamination of fly adhesive pads by microparticles detached from the coatings could additionally decrease the attachment force and contribute to the trapping efficiency of the test tubes (see below).

### 4.2. Effect of Contamination of Insect Attachment Organs by Microparticles

Obtained differences in the contaminating effects of different surface coatings (calcium carbonate, kaolin, and *N. alata* epicuticular wax coverage) were caused primarily by differences in dimensions and shapes of particles composing these coatings. The calcium carbonate coating consists of relatively big, coarse, and robust surface structures, which did not readily detach from the surface under the weight of tiny *D. melanogaster* flies. These particles being rather voluminous and compact did not adhere well to insect adhesive setae and, therefore, did not produce strong contamination of insect adhesive organs. As kaolin particles are smaller and have a flattened shape (resembling wax projections found in a number of *Nepenthes* species [30,33,34]), they detached easily from the substrate, adhered to and heavily contaminated fly setal tips, which are known to be responsible for contact formation during the attachment of insects to smooth or microrough substrates [68]. Similar results for the contaminability of both calcium carbonate and kaolin coatings were obtained previously in the experimental study with the southern green stinkbug *Nezara viridula* (Linnaeus, 1758) (Hemiptera: Pentatomidae) and the Mediterranean fruit fly *Ceratitis capitata* (Wiedemann, 1824) (Diptera: Tephritidae), in which these coatings caused a strong reduction of the insect attachment ability mainly due to the contamination of adhesive pads [58,59]. Interestingly, both contaminating and insect attachment reduction effects were much more pronounced in the case of kaolin. The pear psylla *Cacopsylla pyricola* (Foerster, 1848) (Hemiptera: Psyllidae) also demonstrated a significantly decreased ability to hold onto plant surfaces covered with different types of kaolin coatings [71].

A heavy contamination of *D. melanogaster* attachment organs and especially adhesive pads was observed in our tests with the wax coverage of *N. alata* pitchers bearing microscopic platelet-shaped wax particles. The contaminating ability of *Nepenthes* wax has been repeatedly reported previously [4,21,30,33,36] including the study with *D. melanogaster* flies [36]. The reduction of insect attachment caused by contamination of their adhesive pads with epicuticular plant wax projections has been revealed by both experimental [72,73] and theoretical studies [74,75], showing that this effect is especially pronounced in plant surfaces covered with wax projections having higher aspect ratios.

## 5. Conclusions

Our trapping experiments with differently prepared tubes serving as biomimetic pitchers clearly demonstrated that the type of liquid had the most important impact on the trapping function: surfactant-containing liquids having strong wetting properties in contrast to water, ensured high success in trapping *D. melanogaster* flies. The diameter of the trap rather than its height, which had a relatively small range in our study, affected the trapping efficiency, with wider tubes capturing more insects than narrower ones, probably by providing a larger space for more insects to get into traps. The presence of a microparticle coating mimicking the epicuticular wax coverage inside the pitcher, which is characteristic of numerous *Nepenthes* species, additionally contributed to the high capture achievement, not only due to reduced contact between the trap surface and insect feet but also because of the contamination of insect attachment organs by the particles detached from the coating.

## Figures and Tables

**Table 1:** Results of trapping experiments performed with different tube types using eight set-ups.

Set- Up	Tube Type	Liquid	Tested Character	Categories	Trapped Total ^1^	Trapped Per Tube ^2^	Trapped Per Tube (%) ^3^
1	10 cm high	water	diameter	17 mm	3	0.20	0.07
				30 mm	23	1.53	0.51
2	10 cm high	Triton	diameter	17 mm	59	3.93	1.31
				30 mm	240	16.00	5.36
3	10 cm high	Domol	diameter	17 mm	40	2.67	0.89
				30 mm	241	16.07	5.36
4	30 mm diameter	water	height	8 cm	3	0.33	0.11
				9 cm	5	0.56	0.19
				10 cm	1	1.11	0.04
5	30 mm diameter	Triton	height	8 cm	52	5.78	1.93
				9 cm	61	6.78	2.26
				10 cm	129	14.33	4.78
6	30 mm diameter	Domol	height	8 cm	97	10.78	3.59
				9 cm	86	9.56	3.19
				10 cm	108	12.00	4.00
7	10 cm high, 30 mm diameter	Triton	calcium carbonate coating	no	125	8.33	2.78
				yes	175	11.67	3.89
8	10 cm high, 30 mm diameter	Domol	kaolin coating	no	63	10.50	3.50
				yes	233	38.83	12.94

^1^ *Trapped total* is the number of *Drosophila melanogaster* individuals trapped by all tubes of this type in three experimental boxes. ^2^ *Trapped per tube* is an average number of trapped flies per tube of this type (calculated as number of flies trapped by all tubes of this type divided by number of tubes of this type). ^3^ *Trapped per tube (%)* is an average number of trapped flies per tube of this type calculated in relation to 300 fly individuals initially inserted into the 3 boxes.

## Data Availability

Data are contained within the article. Further raw data will be made available by the authors upon request.

## Abbreviations

The following abbreviations are used in this manuscript:

CA	Contact angle
SEM	Scanning electron microscopy
